# Synthesis and Properties of Semi-Alicyclic Polyimides Based on an Asymmetric Diamine from Renewable Menthofuran

**DOI:** 10.3390/polym18212771

**Published:** 2026-11-06

**Authors:** Lei Xiong, Xuwen Sun, Tao Yuan, Bing Xiong, Yanxiang Mao, Haixia Qi, Feng Liu

**Affiliations:** 1 School of Pharmacy, Nanchang Medical College, Nanchang 330052, China; xionglei@ncmc.edu.cn (L.X.); sunxuwen@ncmc.edu.cn (X.S.); yuantao@ncmc.edu.cn (T.Y.); 2 College of Science and Technology, Nanchang University, Gongqingcheng 332020, China; xiongbing@ncuky.edu.cn; 3 School of Chemistry and Chemical Engineering, Nanchang University, Nanchang 330031, China; myx_ncu@163.com

**Keywords:** polyimide, menthofuran, semi-alicyclic diamine, non-coplanar, asymmetric, charge transfer complexes

## Abstract

Conventional aromatic polyimides suffer from poor solubility, high water absorption and low visible-light transmittance due to strong interchain charge transfer complex (CTC) interactions, limiting their microelectronic and optoelectronic applications. Here, a novel asymmetric bio-based diamine ENDAP bearing a 3,4,5,6,7,8-hexahydro-2H-2,4a-epoxynaphthalene-3,4-diyl (HEN) alicyclic skeleton was synthesized from menthofuran via a four-step route. ENDAP was polymerized with five commercial dianhydrides to give polyimide films. GPC gave M_n_ of 3.17–4.68 × 10^4^ and M_w_ of 4.51–7.94 × 10^4^ Da. These amorphous polyimides, with d-spacings of 4.70–5.49 Å, were mostly soluble in polar aprotic solvents. Water contact angles were 83.3–98.6°, with a minimum water absorption of 0.36% after 48 h. T_g_ ranged from 164 to 211 °C, the maximum T_10_ was 451 °C under N_2_, cutoff wavelengths were 353–388 nm, and maximum T_450_ of 83.6% and maximum T_500_ of 86.1%. The HEN skeleton contributes to improved solubility, reduced water absorption and enhanced transparency, likely by weakening CTC interactions and loosening chain packing, as reflected by the enlarged interchain d-spacing, although the extent depends on the dianhydride structure. This work suggests that renewable semi-alicyclic polyimides are potential candidates for microelectronic and optoelectronic applications.

## 1. Introduction

Polyimides (PIs) are widely deployed in aerospace, microelectronics, flexible displays and high-frequency communication fields owing to their excellent thermal stability, mechanical strength and electrical insulation properties [1,2]. Nevertheless, conventional aromatic polyimides possess rigid backbones and exhibit strong interchain charge transfer complex (CTC) interactions. These intrinsic features cause poor solubility, difficult processing, considerable water absorption and insufficient visible light transparency, severely limiting their application in high-end microelectronic and optoelectronic devices [3,4]. To address these drawbacks, two major modification routes have been established: physical modification and chemical modification.

For physical modification, thermal conductivity, water resistance and optical performance can be improved by incorporating nanofillers, lamellar barrier fillers, porous architectures, or transparent nanocomposites [5,6,7,8]. However, nanofillers tend to aggregate and form defects, porous structures increase water absorption and reduce mechanical strength, and lamellar fillers sacrifice transparency. These approaches usually improve only part of the performance and require complex fabrication.

To circumvent these inherent limitations of physical composites, chemical modification enables intrinsic molecular level tuning of chain packing behavior, fractional free volume, polarity, and interchain CTC interactions by introducing flexible groups, bulky side substituents, fluorinated moieties, alicyclic/aliphatic units and non-coplanar/twisted skeletons. Among these structural motifs, bulky alicyclic segments, asymmetric or non-coplanar architectures, together with flexible ether or ester-based linkages can act individually or produce synergistic effects, rendering this combined design strategy particularly attractive. To begin with, alicyclic units disrupt backbone conjugation and suppress CTC formation, improving transparency and reducing water absorption. Zhou [9] reported biphenyl-fused alicyclic dianhydride-based PIs with T_g_ up to 377 °C, λ_c_ of 315 nm, and T_450_ of 82%. Xiong [10] prepared norbornene-containing PIs with T_g_ of 191–231 °C, λ_c_ of 376–471 nm, visible transmittance of 56–87%, and water absorption of 0.26–0.55%. Next, non-coplanar and asymmetric structures loosen chain stacking and enlarge free volume. She [11] obtained twisted triarylmethane PIs with T_g_ of 319–330 °C, λ_c_ of 335–360 nm, T_450_ of 67–81%, and water absorption of 1.20–1.98%. Zhu [12] reported benzimidazolone-based non-coplanar PIs with T_g_ of 346–402 °C, λ_c_ of 332–393 nm, maximum transmittance of 88.6%, and water absorption of 0.23–2.93%. Furthermore, flexible ether or ester linkages improve processability while retaining backbone rigidity. Yu [13] demonstrated ether/ketone thermoplastic PIs with T_g_ of 184–225 °C, λ_c_ of 364–396 nm, T_450_ of about 84–88%, and water absorption of 0.27–0.89%. Zhang [14] reported main chain ester-containing polyimides with T_g_ above 480 °C and water absorption below 0.83%.

Bio-renewable alicyclic or heterocyclic monomers have attracted attention for balancing performance and sustainability. Furan-2,5-dicarboxylic acid and its derivatives are well-known bio-based building blocks, and menthol has a rigid chiral cyclohexane skeleton that can modulate chain packing [15,16,17,18]. Menthofuran, a monoterpene with a 4,5,6,7-tetrahydrobenzofuran skeleton, is biosynthesized from pulegone in peppermint, and its stereoselective formation has been studied [19]. Menthol can be converted into menth-2-ene oxide and copolymerized with CO_2_ to give the biobased polycarbonate with high thermal stability [20]. However, menthofuran has not yet been used as a building block for polyimides. Its asymmetric, oxygen-containing fused-ring skeleton is expected to disrupt chain packing and suppress CTC interactions, thereby improving solubility and optical transparency. Menthofuran meets green chemistry and carbon-neutrality goals, and its asymmetric skeleton broadens the design space for functional polymers.

Against this background, a diamine monomer (ENDAP) containing the 3,4,5,6,7,8-hexahydro-2H-2,4a-epoxynaphthalene-3,4-diyl (HEN) skeleton and pendant methyl groups was synthesized from menthofuran, maleic anhydride, and 4-fluoronitrobenzene. Although the route involves four steps, it was deliberately designed rather than selected for convenience: all steps use readily available commercial reagents under mild conditions (≤110 °C, ambient pressure) and require only recrystallization/filtration for purification, and each step is structurally indispensable, with the Diels–Alder reaction fixing the rigid, non-coplanar HEN skeleton, selective anhydride reduction providing the two hydroxymethyl arms, and etherification/reduction installing the para-aniline termini required for polycondensation. To our knowledge, no commercially available simple diamine offers the combination of the fused oxabicyclic HEN unit and two methyl substituents, and because all steps proceed under mild conditions and require only simple purification, the laboratory-scale route is amenable to preliminary scale-up. The HEN skeleton and pendant methyl groups disrupt backbone conjugation and suppress intra- and intermolecular CTC formation, while the bulky, rigid, non-coplanar architecture increases interchain free volume and inhibits dense chain packing; together with the flexible ether linkages that enhance segmental mobility, these features are expected to improve solubility, solution-casting processability, hydrophobicity and optical transparency. To examine whether these structural features translate into the expected properties, ENDAP was polymerized with five commercial dianhydrides (BPDA, 6FDA, ODPA, BTDA, and BPADA) to produce a series of polyimide films, which were systematically characterized for thermal stability, solubility, hydrophobicity and optical performance to elucidate the structure–property relationships.

## 2. Materials and Methods

### 2.1. Materials

Menthofuran (≥98%), maleic anhydride (≥99%), lithium aluminum hydride (LAH, ≥97%, powder), palladium on carbon (10% Pd, wet), 4-fluoronitrobenzene (≥99%) and sodium hydride (NaH, 60% dispersion in mineral oil) were purchased from Shanghai Aladdin Biochemical Technology Co., Ltd., Shanghai, China. Tetrahydrofuran (THF), N,N-dimethylformamide (DMF), N,N-dimethylacetamide (DMAc, anhydrous), toluene (TL), 80% hydrazine hydrate, trichloromethane (CF), dimethyl sulfoxide (DMSO), ethyl alcohol (ET), ethyl acetate (EA), dichloromethane (DCM), N-methylpyrrolidone (NMP), 3,3′,4,4′-biphenyltetracarboxylic dianhydride (BPDA, ≥98%), 4,4′-(hexafluoroisopropylidene) diphthalic anhydride (6FDA, ≥98%), 4,4′-oxydiphthalic anhydride (ODPA, ≥98%), 3,3′,4,4′-benzophenonetetracarboxylic dianhydride (BTDA, ≥98%) and 4,4′-bisphenol A dianhydride (BPADA, ≥98%) were purchased from Sinopharm Chemical Reagent Co., Ltd., Shanghai, China. Distilled water was prepared in our laboratory. All chemicals were used as received without further purification. Unless otherwise stated, all solvents were of analytical grade.

### 2.2. Characterization

FT-IR spectra (KBr) were recorded on an FTS-40 Fourier transform infrared spectrometer (Thermo Fisher Scientific, Waltham, MA, USA) with a scanning range of 400–4000 cm^−1^. ^1^H-NMR and ^13^C-NMR spectra were measured on a DRX300 spectrometer (Bruker, Karlsruhe, Germany) with tetramethylsilane (TMS) as internal standard, and DMSO-*d*_6_ as solvent. TGA was performed using a Q500 thermogravimetric analyzer (TA Instruments, New Castle, DE, USA) at a heating rate of 20 °C·min^−1^ in flowing nitrogen over 40–800 °C. Approximately 10 mg of sample was used for each test. DSC was performed using a DSC 214 Polyma differential scanning calorimeter (Netzsch, Selb, Germany) at a heating/cooling rate of 20 °C·min^−1^ in flowing nitrogen over 50–370 °C. The samples were subjected to a heating–cooling–heating cycle, and the glass transition temperatures (T_g_) were determined from the second heating scan. Number-average molecular weight (M_n_) and weight-average molecular weight (M_w_) of PI films were determined on a Waters E2695 gel permeation chromatography (GPC) system (Waters, Milford, MA, USA) equipped with a refractive index detector. Two mixed-bed organic GPC columns covering a molecular weight range of 10^2^–10^6^ g·mol^−1^ were used. NMP containing 0.03 mol·L^−1^ LiBr and 0.03 mol·L^−1^ H_3_PO_4_ was used as the eluent at a flow rate of 1.0 mL·min^−1^, with the column temperature maintained at 40 °C. The sample concentration was 1.0 mg·mL^−1^, and the injection volume was 100 μL. Polystyrene standards with molecular weights ranging from approximately 580 to 1,000,000 g·mol^−1^ were used for calibration. WAXD measurements were performed using a SmartLab 9KW diffractometer (Rigaku, Tokyo, Japan) with Cu/Kα radiation (λ = 0.154 nm) over 2θ = 5–55°. Contact angle measurements were performed using a CA200 contact angle instrument (Guangdong Beidou Precision Instrument Co., Ltd., Dongguan, China) with deionized water as the probe liquid, and the data are reported as the average of five replicate measurements. The thickness of PI films was measured on a Fischer DUALSCOPE FMP40 thickness gauge (Fischer Sindelfingen, Technology, Germany). UV–Vis spectra were recorded on a 1800PC spectrophotometer (Shanghai Mapada Instrument Co., Ltd., Shanghai, China) over 300–800 nm at room temperature. Melting points were measured on a JH80 automatic video melting point tester (Shanghai Precision Instrumentation Co., Ltd., Shanghai, China) using a two-stage heating program: 20 °C·min^−1^ initially, reduced to 1.0 °C·min^−1^ when the temperature was about 15 °C below the expected melting point. The instrument was calibrated with a benzoic acid standard prior to measurement. For all measurements, values are reported together with the accuracy of their determination: melting points ± 0.3 °C; temperatures from DSC and TGA (T_g_, T_5_, T_10_ and T_max_) ± 1 °C; water contact angles ± 0.1°; number- and weight-average molecular weights ± 5% (relative); interchain d-spacing from WAXD ± 0.01 Å; and film thickness ± 1 μm. Molecular weights are quoted to three significant figures to allow direct comparison within the series, and contact angles to 0.1°.

### 2.3. Molecular Simulation and Quantum Chemical Calculations

Molecular simulations were performed using Materials Studio 2020 (BIOVIA, San Diego, CA, USA). Amorphous cell models of PI 1–5 and Kapton were constructed within the Amorphous Cell module. Polymer chains containing 10 repeating units were built and geometrically optimized using the Forcite module with the COMPASS II force field, and then randomly packed into cubic amorphous cells of 4.1 × 4.1 × 4.1 nm^3^. Periodic boundary conditions were applied. After energy minimization, the systems first underwent NPT ensemble molecular dynamics (MD) at 0.1 GPa for 40 ps, followed by NPT relaxation for 500 ps. The cells were subsequently annealed from 300 K to 700 K under the NPT ensemble for five cycles with a total simulation time of 800 ps, and subjected to an additional 500 ps NPT relaxation. A final 700 ps NVT simulation was carried out. To obtain structures corresponding to ambient-condition films, each system was further equilibrated under the NPT ensemble at 298 K and 0.1 MPa. The Berendsen barostat and Nosé thermostat were adopted throughout all MD stages for pressure and temperature control. After full equilibration at 298 K and 0.1 MPa, the density and fractional free volume (FFV) of each cell were calculated via the Connolly surface method with a 0.1 nm probe radius. FFV is defined as FFV = V_free_/(V_free_ + V_occupied_), where V_free_ and V_occupied_ correspond to the free volume and occupied volume of the simulation system, respectively. High-pressure compression and high-temperature annealing serve only as numerical relaxation strategies for eliminating nonphysical artifacts generated during cell construction, and do not replicate experimental thermal treatment conditions. All structural properties were sampled under ambient condition equilibration.

Density functional theory (DFT) calculations were carried out on individual polyimide repeating units. Molecular models were constructed in ChemDraw 3D 2021 21.0 (Revvity Signals Software, Waltham, MA, USA) and geometrically optimized using Gaussian 16 A.03 (Gaussian, Inc., Wallingford, CT, USA) with the 6-31G(d,p) basis set. The highest occupied molecular orbital (E_HOMO_), lowest unoccupied molecular orbital (E_LUMO_), and molecular orbital energy gap (ΔE = E_LUMO_ − E_HOMO_) were extracted from the optimized structures.

### 2.4. Synthesis of the Diamine Monomer

**Scheme 1:**
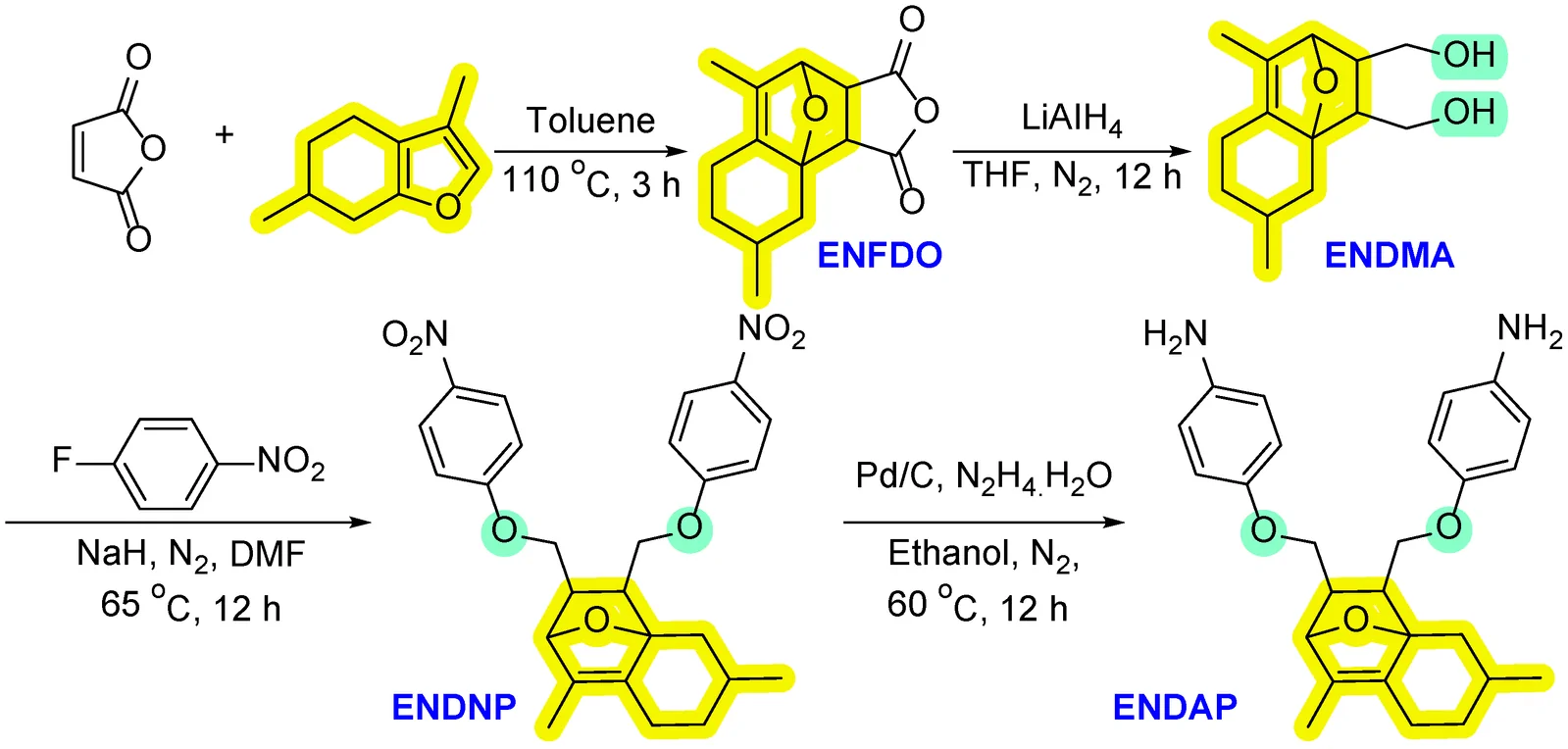
Synthesis route of ENDAP.

4,4′-(((1,6-dimethyl-3,4,5,6,7,8-hexahydro-2H-2,4a-epoxynaphthalene-3,4-diyl)bis(methylene))bis(oxy))dianiline (ENDAP) was synthesized in four steps, as shown in Scheme 1. Maleic anhydride and menthofuran first underwent Diels–Alder cycloaddition to give a cyclic anhydride bearing a rigid HEN skeleton. Selective LAH reduction gave the diol, which was then reacted with 4-fluoronitrobenzene via nucleophilic substitution to form a dinitro compound with flexible ether linkages. Pd/C-catalyzed reduction of this dinitro intermediate finally gave the target diamine monomer, containing both the rigid HEN skeleton and flexible ether bonds.

#### 2.4.1. Synthesis of 5,8-Dimethyl-3a,6,7,8,9,9b-hexahydro-3H-4,9a-epoxynaphtho[1,2-c]furan-1,3(4H)-dione (ENFDO)

Maleic anhydride (1.5013 g, 0.0153 mol) and toluene (15 mL) were added into a 100 mL three-neck flask equipped with a reflux condenser, and heated to 110 °C with stirring until complete dissolution. Menthofuran (2.3001 g, 0.0153 mol) was then added rapidly, causing the mixture to turn dark green. After stirring at 110 °C for 3 h, the mixture turned wine-red. Upon cooling to room temperature, a large amount of solid precipitated. The precipitate was collected by filtration, recrystallized from toluene, and dried under vacuum at 60 °C to afford ENFDO as white needle-like crystals. Yield: 71.97%; m.p.: 142.6–143.9 °C. FT-IR, ν/cm^−1^: 1856(C=O), 1780(C=O), 1679(C=C); ^1^H-NMR (300 MHz, DMSO-*d*_6_), δ: 4.90 (s, 1H), 3.41 (d, J = 5.1, 1H), 3.24 (d, J = 4.8, 1H), 2.42 (dd, J_1_ = 12.6, J_2_ = 2.4, 2H), 2.15 (d, J = 9.6, 2H), 1.96–1.83 (m, 2H), 1.68 (d, J = 1.5, 3H), 1.39 (t, J = 9.6, 1H), 0.98 (d, J = 4.8, 3H); ^13^C-NMR (75 MHz, DMSO-*d*_6_), δ: 172.19, 170.62, 139.90, 136.62, 90.79, 84.15, 53.11, 50.35, 34.81, 32.93, 28.79, 22.49, 21.64, 10.13.

#### 2.4.2. Synthesis of (1,6-Dimethyl-3,4,5,6,7,8-hexahydro-2H-2,4a-epoxynaphthalene-3,4-diyl)dimethanol (ENDMA)

LAH (0.9198 g, 0.0242 mol) and THF (15 mL) were added to a 100 mL three-neck flask cooled in an ice bath under N_2_ atmosphere. A solution of ENFDO (3.0013 g, 0.0121 mol) in THF (25 mL) was then slowly added dropwise, and the mixture was stirred at room temperature for 12 h. Distilled water was added dropwise to quench the unreacted LAH, and the mixture was filtered. The filtrate was concentrated on a rotary evaporator under water-aspirator vacuum (vacuum degree 0.095 MPa) at a bath temperature of 25 °C to afford a dark red viscous residue, which was taken up in THF at 60 °C and stirred for 30 min. After filtration to remove insoluble impurities, the filtrate was concentrated to a small volume under the same conditions. Distilled water was added to cover the liquid surface, and the mixture was stored in a refrigerator for 12 h. White needle-like crystals of ENDMA precipitated, which were collected by filtration and dried under vacuum at 30 °C for 6 h. Yield: 62.12%; m.p.: 61.3–63.0 °C. FT-IR, ν/cm^−1^: 3266(O-H), 1660(C=C); ^1^H-NMR (300 MHz, DMSO-*d*_6_), δ: 4.64 (t, J = 2.7, 2H), 4.49 (t, J = 2.4, 2H), 4.26 (s, 1H), 3.62–3.58 (m, 1H), 3.23 (td, J_1_ = 4.8, J_2_ = 2.7, 1H), 2.39 (dd, J_1_ = 8.1, J_2_ = 1.5, 2H), 2.04 (d, J = 6.3, 2H), 1.85–1.80 (m, 2H), 1.59 (d, J = 0.6, 3H), 1.20 (t, J = 6.3, 1H), 0.92 (d, J = 3.3, 3H); ^13^C-NMR (75 MHz, DMSO-*d*_6_), δ: 139.04, 133.99, 88.95, 82.17, 60.84, 59.43, 46.57, 43.77, 36.50, 33.69, 29.24, 22.88, 22.19, 10.12.

#### 2.4.3. Synthesis of 1,6-Dimethyl-3,4-bis((4-nitrophenoxy)methyl)-3,4,5,6,7,8-hexahydro-2H-2,4a-epoxynaphthalene (ENDNP)

NaH (0.8311 g, 0.0346 mol) was added to a 100 mL three-neck flask equipped with a reflux condenser under N_2_ atmosphere, and a solution of ENDMA (1.5010 g, 0.0062 mol) in DMF (20 mL) was added dropwise. The mixture was stirred at 65 °C for 3 h and then cooled to room temperature. 4-fluoronitrobenzene (1.8004 g, 0.0125 mol) was added dropwise, turning the mixture brownish yellow. After stirring at room temperature for 12 h, distilled water was added dropwise until a large amount of solid precipitated. The solid was collected by filtration, recrystallized from DMF, and dried under vacuum at 80 °C for 12 h to afford ENDNP as a yellow solid. Yield: 76.62%; m.p.: 162.0–163.2 °C. FT-IR, ν/cm^−1^: 1606(C=C), 1485(N=O), 1331(N=O), 1117(C-O), 1067(C-O), 820(C-N); ^1^H-NMR (300 MHz, DMSO-*d*_6_), δ: 8.27 (s, 2H), 8.10 (s, 2H), 7.26 (d, J = 6.4, 2H), 6.81 (s, 2H), 4.49 (s, 2H), 4.40 (s, 2H), 4.16 (d, J = 16.5, 1H), 3.39 (s, 1H), 3.14 (s, 1H), 2.36 (s, 2H), 2.23 (s, 2H), 1.96 (s, 2H), 1.71 (s, 3H), 1.40–1.28 (m, 1H), 0.96 (s, 3H); ^13^C-NMR (75 MHz, DMSO-*d*_6_), δ: 164.30, 163.94, 141.44, 139.17, 134.36, 126.36, 126.30, 115.73, 115.41, 89.34, 82.05, 68.88, 67.72, 43.69, 41.07, 36.56, 33.50, 29.29, 22.77, 22.21, 10.14.

#### 2.4.4. Synthesis of 4,4′-(((1,6-Dimethyl-3,4,5,6,7,8-hexahydro-2H-2,4a-epoxynaphthalene-3,4-diyl)bis(methylene))bis(oxy))dianiline (ENDAP)

ENDNP (4.2931 g, 0.0089 mol), Pd/C (0.1297 g) and anhydrous ethanol (50 mL) were added to a 150 mL three-neck flask equipped with a reflux condenser and heated to 60 °C under N_2_ atmosphere. Hydrazine hydrate (25 mL, 80%) was then added dropwise, and the mixture was stirred at 60 °C for 12 h. The mixture was filtered three times, and the filtrate was subsequently concentrated on a rotary evaporator under water-aspirator vacuum (vacuum degree 0.095 MPa) at a bath temperature of 35 °C until abundant solid precipitated. The solid was collected by filtration, recrystallized from anhydrous ethanol, and dried under vacuum at 35 °C for 10 h to afford ENDAP as a white solid. Yield: 62.21%; m.p.: 151.0–152.8 °C. FT-IR, ν/cm^−1^: 3431(N-H), 3347(N-H), 1633(C=C); ^1^H-NMR (300 MHz, DMSO-*d*_6_), δ: 6.65 (dd, J_1_ = 12.6, J_2_ = 6.3, 4H), 6.48 (d, J = 5.4, 4H), 4.58 (d, J = 6.6, 4H), 4.35 (s, 1H), 4.02–3.96 (m, 2H), 3.73–3.67 (m, J = 8.4, 2H), 2.41 (dd, J_1_ = 11.7, J_2_ = 2.1, 2H), 2.14–2.08 (m, 2H), 2.00–1.83 (m, 2H), 1.69 (d, J = 7.8, 2H), 1.61 (s, 3H), 1.23 (t, J = 9.3, 1H), 0.90 (d, J = 4.8, 3H); ^13^C-NMR (75 MHz, DMSO-*d*_6_), δ: 150.24, 143.02, 139.05, 134.17, 116.23, 115.30, 89.29, 82.11, 68.40, 66.50, 43.76, 41.07, 36.50, 33.54, 29.23, 22.78, 22.21, 10.16.

### 2.5. Film Preparation

**Scheme 2:**
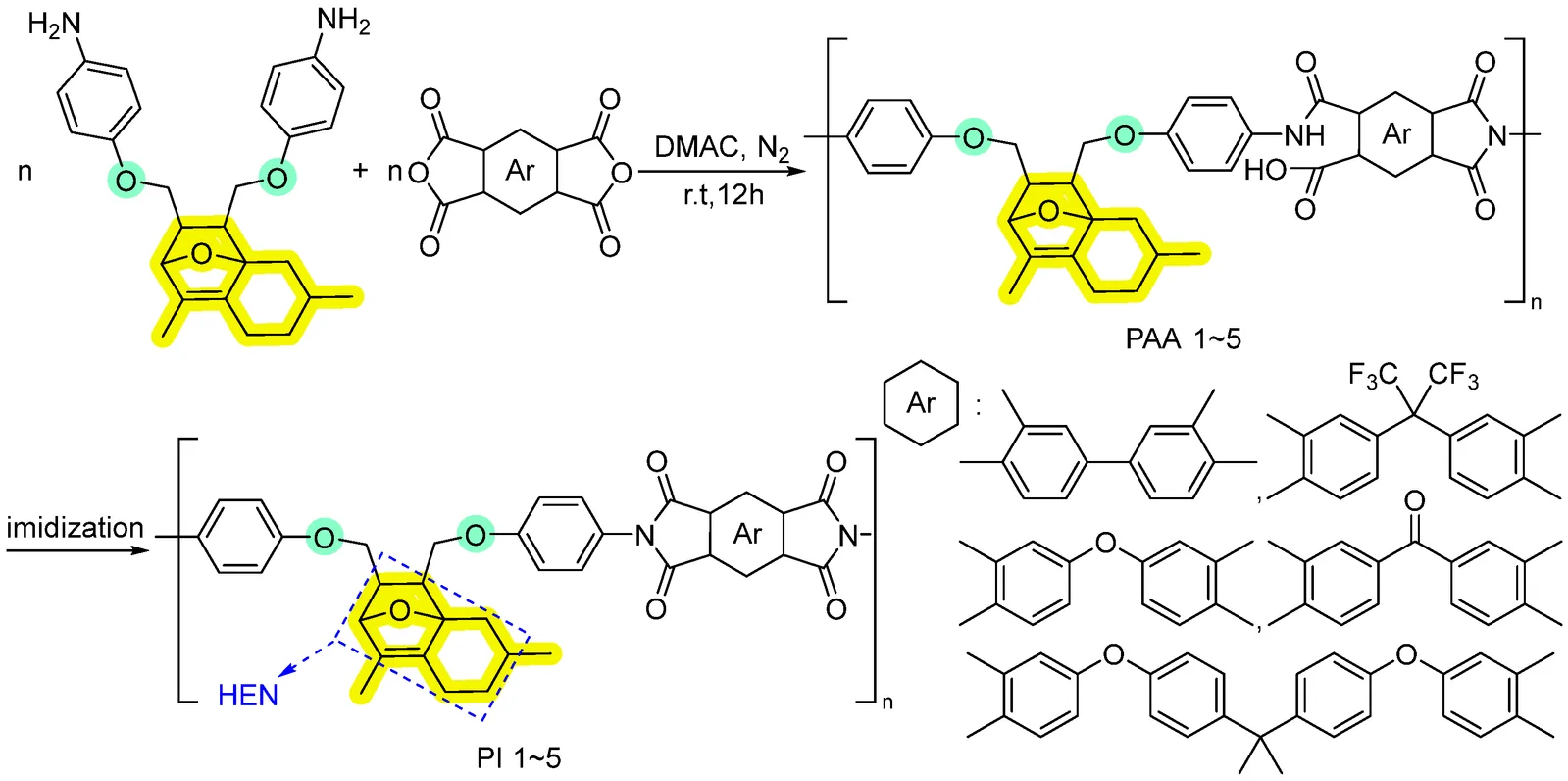
Synthesis route of PI films.

**Figure 1:**
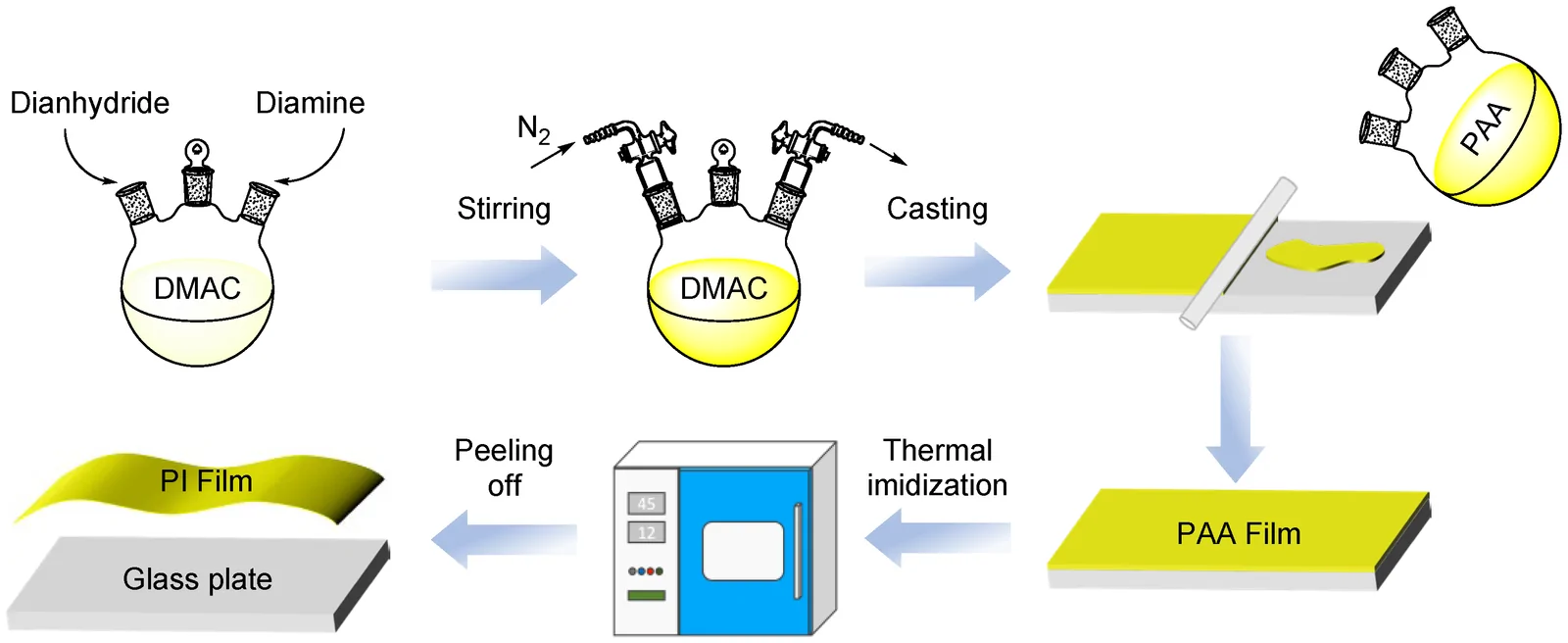
Schematic of the PI films fabrication process.

Polyimides were synthesized from ENDAP and each of the five commercial dianhydrides via the synthetic route shown in Scheme 2, and the corresponding films were prepared from the resulting PAA solutions as illustrated in Figure 1. A typical procedure for PI 1 (ENDAP-BPDA) is given below. ENDAP (1.0001 g, 0.0024 mol) and BPDA (0.7061 g, 0.0024 mol) were added in an exactly equimolar ratio to a 25 mL three-neck flask with anhydrous DMAc (solid content of 30 wt%) and stirred under N_2_ for 12 h to afford a viscous pale yellow poly(amide acid) solution (PAA). The PAA was then uniformly cast onto a 5 × 10 cm glass plate, thermally imidized at 60 and 80 °C for 2 h each, followed by 100, 140, 180, and 230 °C for 1 h each. Temperatures above 230 °C caused noticeable film darkening, so the final imidization temperature was kept at 230 °C. The resulting PI film was peeled off the glass substrate using distilled water. PI 2 (ENDAP-6FDA), PI 3 (ENDAP-ODPA), PI 4 (ENDAP-BTDA), and PI 5 (ENDAP-BPADA) were prepared following the same procedure. The thickness of the resulting films, measured with the thickness gauge described in Section 2.2, was 68–82 μm.

PI 1 (ENDAP-BPDA). FT-IR, ν/cm^−1^: 2922, 1773(C=O), 1718(C=O), 1609(C=C), 1508, 1458, 1375(C-N), 1296, 1222, 1171, 1114, 1085, 1010, 825, 737.

PI 2 (ENDAP-6FDA). FT-IR, ν/cm−1: 3077, 2924, 1784(C=O), 1724(C=O), 1611(C=C), 1510, 1458, 1380(C-N), 1298, 1254, 1145, 1097, 1017, 983, 826, 722.

PI 3 (ENDAP-ODPA). FT-IR, ν/cm^−1^: 2921, 1773(C=O), 1717(C=O), 1607(C=C), 1508, 1473, 1375(C-N), 1298, 1237, 1174, 1088, 821, 744.

PI 4 (ENDAP-BTDA). FT-IR, ν/cm^−1^: 3065, 2925, 1777(C=O), 1719(C=O), 1604(C=C), 1508, 1458, 1375(C-N), 1241, 1172, 1118, 1093, 1019, 936, 820, 712;

PI 5 (ENDAP-BPADA). FT-IR, ν/cm^−1^: 2924, 2854, 1777(C=O), 1714(C=O), 1597(C=C), 1510, 1475, 1377(C-N), 1272, 1236, 1170, 1073, 1012, 823, 740.

## 3. Results and Discussion

### 3.1. The Structural Characterization of Monomer

**Figure 2:**
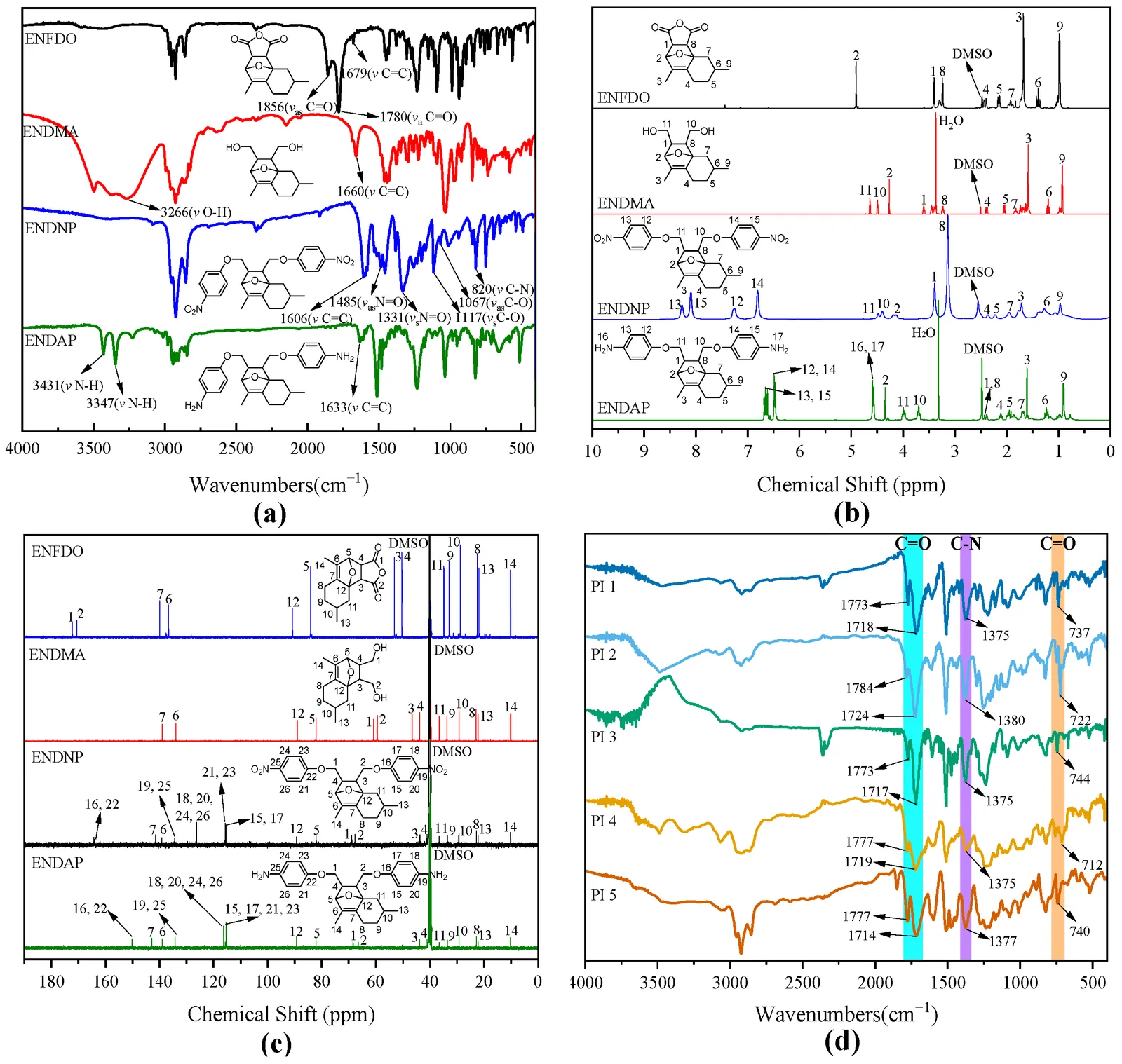
(**a**) FT-IR spectra, (**b**) ^1^H-NMR spectra, (**c**) ^13^C-NMR spectra of ENFDO, ENDMA, ENDNP and ENDAP; (**d**) FT-IR spectra of PI 1–5.

The chemical structures of ENDAP and its intermediates were characterized using FT-IR, ^1^H-NMR, and ^13^C-NMR spectroscopy, with their corresponding spectra presented in Figure 2a, b, and c, respectively.

As shown in Figure 2a, the distinct bands of ENFDO at 1856 and 1780 cm^−1^ are assigned to the asymmetric and symmetric C=O stretching vibrations of the anhydride, respectively. The band at 1679 cm^−1^, corresponding to endocyclic C=C stretching, indicates that the Diels-Alder reaction between menthofuran and maleic anhydride has occurred. In the ENDMA spectrum, the anhydride carbonyl bands at 1856 and 1780 cm^−1^ disappear completely, while a prominent broad band attributable to O-H stretching appears at 3200–3500 cm^−1^, indicating reduction of the anhydride groups to hydroxyl groups. Compared with ENDMA, the broad hydroxyl absorption of ENDNP at 3200–3500 cm^−1^ vanishes entirely, and two strong characteristic nitro-group bands are observed at 1485 and 1331 cm^−1^, confirming the formation of the target dinitro product. In the ENDAP spectrum, characteristic N-H stretching bands are observed at 3431 and 3347 cm^−1^, indicating reduction of the nitro groups to amino moieties.

As can be seen from the ENDAP spectrum in Figure 2b, peaks 16 and 17 (δ 4.58) are assigned to the hydrogens on amino groups; peaks 12, 13, 14, and 15 (δ 6.65 and 6.48) are assigned to the hydrogen atoms on the benzene ring; peaks 10 (δ 4.02–3.96) and 11 (δ 3.73–3.67) are assigned to the methylene hydrogens bridging the ether linkage and the HEN skeleton; peak 9 (δ 0.90) is assigned to the methyl hydrogens on the saturated six-membered ring; peaks 1 and 8 (δ 2.41) are assigned to the methine hydrogens on the unsaturated six-membered ring; peaks 4 (δ 2.14–2.08), 5 (δ 2.00–1.83), and 7 (δ 1.69) are assigned to the methylene hydrogens on the saturated six-membered ring; peak 6 (δ 1.23) is assigned to the methine hydrogen on the saturated six-membered ring; peak 3 (δ 1.61) is assigned to the methyl hydrogen attached to the double bond; and peak 2 (δ 4.35) is assigned to the hydrogen on the bridgehead carbon of the HEN skeleton.

Consistent with the ^1^H-NMR results, the ^13^C-NMR spectra further confirmed the proposed structures. As can be seen from the ENDAP spectrum in Figure 2c, peaks 4 (δ 41.07) and 3 (δ 43.76) are assigned to the methine carbons on the unsaturated six-membered ring connected to methylene groups; and peaks 5 (δ 82.11) and 12 (δ 89.29) are assigned to the two bridge carbons on the saturated six-membered ring. The peaks at δ 66.50 and δ 68.40 are assigned to the methylene carbons connected to the ether bond. Due to their connection with oxygen, their chemical shifts move to a lower field. Peaks at δ 150.24, δ 143.02, δ 116.23, and δ 115.30 are attributed to the aromatic carbons of the 4-aminophenoxy groups; and the peaks at δ 139.05 and δ 134.17 are attributed to the two carbons of the C=C bond.

### 3.2. Chemical Structure and Aggregated Structure of PI Films

**Figure 3:**
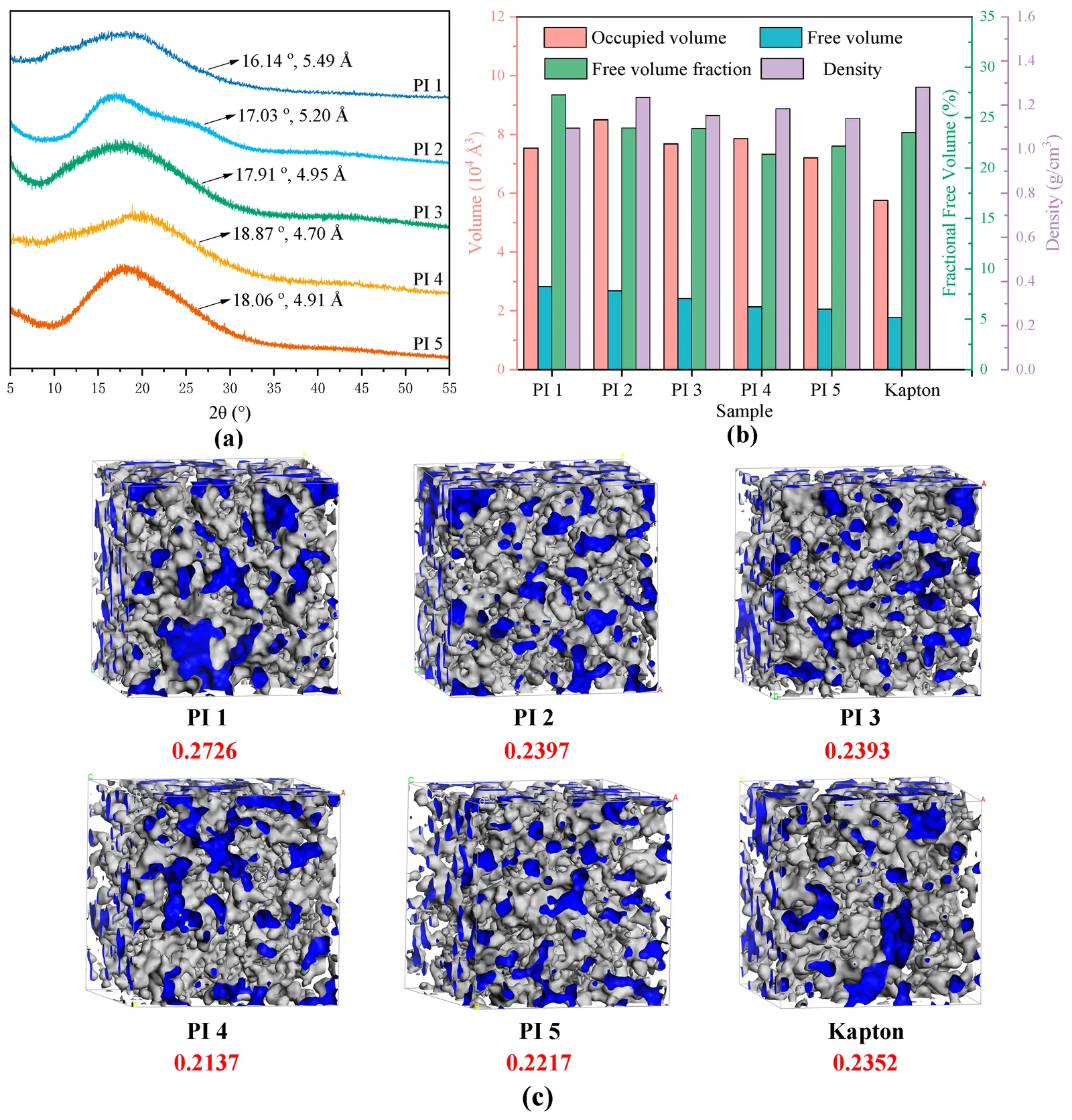
(**a**) WAXD patterns of PI films; (**b**) occupied volume, free volume, fractional free volume, and density for PI films and Kapton; (**c**) molecular dynamics simulated polymer configurations for PI films and Kapton.

The chemical structures, chain packing characteristics and molecular weights of PI films were characterized by FT-IR, WAXD and GPC. The FT-IR spectra are presented in Figure 2d, WAXD patterns in Figure 3a, while relevant WAXD parameters and molecular weight values are summarized in Table 1. GPC measurements reveal that the PI films possess M_n_ and M_w_ of 3.17 × 10^4^–4.68 × 10^4^ Da and 4.51 × 10^4^–7.94 × 10^4^ Da, respectively, with polydispersity index values of 1.36–1.70. The results indicate that the diamine ENDAP possesses sufficient polycondensation reactivity toward all the dianhydrides.

As can be seen in Figure 2d, the bands near 1775 and 1720 cm^−1^ are assigned to asymmetric and symmetric C=O stretching vibrations of the imide carbonyl groups, respectively, whereas the band near 1375 cm^−1^ is attributed to C-N stretching of the imide ring. In addition, the bands at 712–744 cm^−1^ for PI 1–5 are assigned to imide ring deformation. The absence of N-H stretching bands of amino groups in the 3300–3500 cm^−1^ region, of the characteristic anhydride C=O band around 1850 cm^−1^, and of the amide bands at 1650 and 1550 cm^−1^ indicates successful formation of the polyimide and essentially complete imidization. Figure 3a shows broad peaks in all WAXD patterns, indicating that these polyimides are amorphous. This is likely because the asymmetric HEN skeleton disrupts chain ordering and inhibits crystallization. The average interchain spacing (d-spacing) values of the PI films range from 4.70 to 5.49 Å, all of which are higher than the 4.19 Å value for Kapton [21]. The non-coplanar HEN skeleton, together with its pendant methyl groups, is expected to reduce the conjugation of the polymer backbone and to weaken intermolecular charge-transfer (CT) interactions, while its steric hindrance inhibits tight chain packing and thereby enlarges the interchain d-spacing. Direct comparisons using the same dianhydride but different diamines are available for 6FDA and ODPA [12]. For 6FDA, the polyimides derived from the aromatic diamines AHDB, AHFDB and AFDB show d-spacing values of 3.91, 4.30 and 4.19 Å, respectively, all smaller than that of PI 2 (ENDAP-6FDA, 5.20 Å); for ODPA, the corresponding values are 3.57, 4.26 and 4.18 Å, again smaller than that of PI 3 (ENDAP-ODPA, 4.95 Å). These comparisons suggest that, compared with aromatic diamines, the HEN-based diamine contributes to looser chain packing and weakened CTC interactions, although the dianhydride also plays a role.

**Table 1:** Molecular weight and thermal properties of PI films.

Codes	Molecular Weight			Thermal Properties					WAXD	
	M_n_/Da	M_w_/Da	PDI ^a^	T_5_/°C ^b^	T_10_/°C ^c^	T_g_/°C ^d^	T_max_/°C ^e^	Y_800_/% ^f^	2θ/°	d-Spacing/Å ^g^
PI 1	3.21 × 10^4^	4.79 × 10^4^	1.49	361	414	197	440	49.5	16.14	5.49
PI 2	3.17 × 10^4^	4.51 × 10^4^	1.42	374	412	199	464	37.8	17.03	5.20
PI 3	4.22 × 10^4^	5.74 × 10^4^	1.36	431	451	190	462	51.4	17.91	4.95
PI 4	4.43 × 10^4^	6.91 × 10^4^	1.56	341	391	211	415	49.0	18.87	4.70
PI 5	4.68 × 10^4^	7.94 × 10^4^	1.70	392	447	164	511	40.5	18.06	4.91

All thermal measurements were performed under N_2_. ^a^ polydispersity index; ^b,c^ temperatures at 5% and 10% thermal weight loss, respectively; ^d^ glass transition temperature; ^e^ maximum decomposition rate temperature; ^f^ char yield at 800 °C; ^g^ values correspond to the average chain spacing of PIs, which were calculated from the prominent peak of WAXD spectrum by Bragg’s equation [22]: 2dsinθ = nλ, where n is 1, λ is 1.54 Å.

As illustrated in Figure 3b,c, PI 1–3 exhibit higher fractional free volume (FFV) and lower chain packing density than Kapton, whereas PI 4–5 exhibit slightly lower FFV but still lower chain packing density than Kapton. For PI 1 and PI 2, the biphenyl linkage in BPDA and the hexafluoroisopropylidene group in 6FDA introduce rigid, bulky segments that disrupt chain packing. The trifluoromethyl groups in 6FDA further increase side chain volume and hinder close contact between chains, resulting in higher FFV and lower packing density than Kapton. For PI 3, the flexible ether linkage introduces a kink that breaks backbone linearity and creates higher initial free volume, also leading to higher FFV and lower packing density than Kapton. For PI 4 and PI 5, the polar carbonyl bridge in BTDA strengthens intermolecular dipole interactions and promotes tighter chain packing. The isopropylidene group in BPADA introduces steric hindrance that suppresses long range ordered stacking. Meanwhile, its ether linkages endow the backbone with sufficient flexibility to enable more compact local packing relative to ODPA. Both effects reduce FFV relative to Kapton. Nevertheless, these PIs still exhibit lower chain packing density than Kapton, indicating that chain packing density is not solely determined by FFV. This arises from the non-coplanar HEN skeleton that disrupts chain stacking, which simultaneously yields their larger d-spacing compared with Kapton.

### 3.3. Solubility of Diamine and PI Films

To explore the solubility of the diamine and PI films, 100 mg of polymer film fragments were added to 2 mL of each solvent, and the results are summarized in Table 2. The diamine exhibits excellent solubility in various organic solvents. Full dissolution in common polar aprotic polymerization solvents enables low-temperature polycondensation to yield high-molecular-weight PAA precursors for subsequent PI fabrication.

As can be seen from the solubility of PI 1–5 in Table 2, most of these polyimides were soluble in common polar aprotic solvents, while PI 4 was only partially soluble. The bulky, asymmetric HEN skeleton in ENDAP, together with its pendant methyl groups, creates steric hindrance, and its intrinsic asymmetry further disrupts segmental and backbone regularity, thereby inhibiting dense chain packing, suppressing crystallization, and reducing interchain CTC formation, ultimately enhancing solubility [23]. This steric hindrance raises the FFV of the polymer and facilitates penetration of organic solvents into polymer chains. As confirmed by the preceding WAXD results, the same structural modification enlarges interchain d-spacing, which further promotes solvent diffusion. Despite these solubility-favoring structural features originating from ENDAP, the choice of dianhydride still regulates the solubility of the resulting polyimides by altering chain packing, fractional free volume and interchain d-spacing. The bulky hexafluoroisopropylidene group in 6FDA disrupts backbone conjugation and increases free volume and d-spacing, rendering PI 2 the most soluble film in this series. In contrast, the conjugated benzophenone backbone of BTDA facilitates interchain π-π stacking and strengthens intermolecular interactions, restricting solvent diffusion and leaving PI 4 only partially soluble across all tested solvents. For ODPA and BPADA, embedded flexible ether bonds enhance segmental mobility and suppress tight chain packing, improving the solubility of PI 3 and PI 5. Although the rigid biphenyl backbone of BPDA exhibits a tendency for ordered chain stacking, this adverse effect is offset by the high FFV and d-spacing derived from the HEN skeleton of ENDAP. Consistent with the observation in Table 2, PI 1 delivers slightly better solubility than PI 3 and PI 5. Accordingly, PI 1, PI 3, and PI 5 all possess favorable solubility in common organic solvents.

### 3.4. Thermal Properties of PI Films

**Figure 4:**
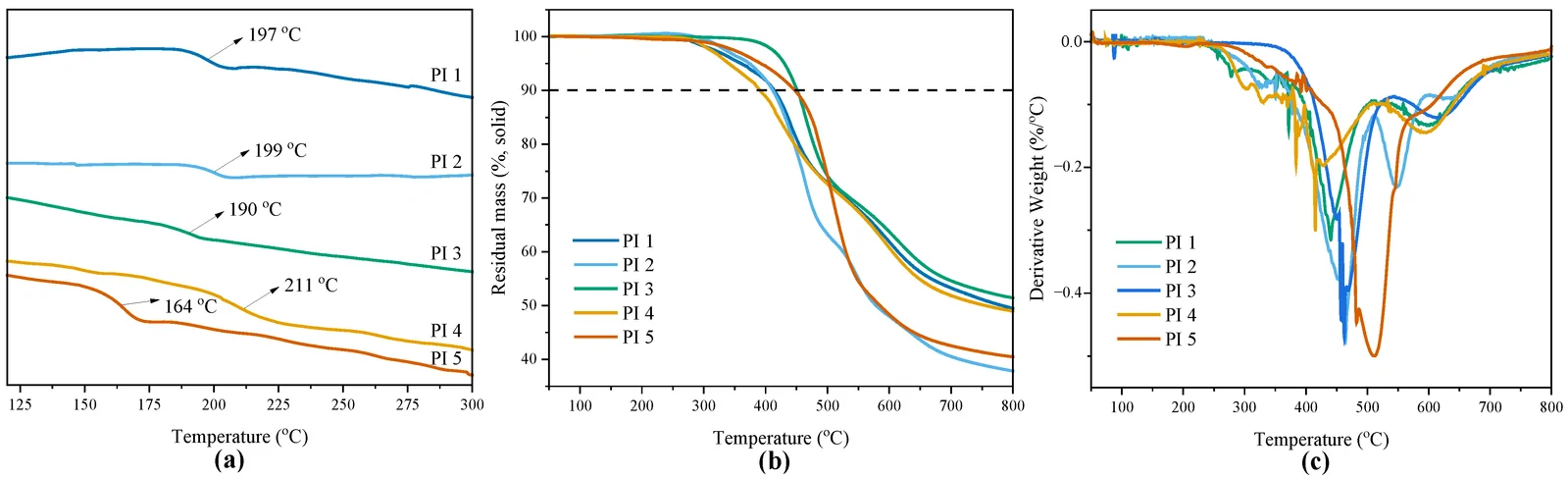
(**a**) DSC, (**b**) TGA and (**c**) DTG curves of PI films.

The thermal properties of PI 1–5 films were characterized using DSC and TGA, with the corresponding DSC, TGA, and DTG curves presented in Figure 4a, b, and c, respectively, where the DTG curves were obtained by differentiating the TGA data. All thermal parameters, including T_g_, 5% weight loss temperature (T_5_), 10% weight loss temperature (T_10_), maximum decomposition rate temperature (T_max_), and char yield at 800 °C (Y_800_) under N_2_ atmosphere, are summarized in Table 1.

T_g_ values of this PI series range from 164 to 211 °C. T_g_ is dictated by backbone rigidity and molecular chain packing characteristics [24], and these properties are substantially modulated by the chemical structures of the selected diamine and dianhydride monomers. The diamine bearing the HEN skeleton introduces bulky, non-coplanar moieties that disrupt dense molecular chain packing and tend to reduce T_g_ [25,26]. Even so, the intrinsic rigid backbone of this diamine allows all the obtained polyimides except PI 5 to maintain T_g_ above 190 °C. Beyond the backbone effect originating from the diamine, flexible ether linkages from different dianhydride structures exert plasticization effects and further tune polymer segmental mobility [27]. Among all samples, PI 4 uses an ether free dianhydride with conjugated planar architecture and exhibits the highest T_g_ in this series. In contrast, PI 3 contains flexible ether linkages from both the diamine and dianhydride, with a T_g_ of 190 °C. PI 5 contains more flexible ether linkages and correspondingly exhibits the lowest T_g_ of 164 °C. These moderate T_g_ values endow the PIs with good thermal processability.

The thermal decomposition data summarized in Table 1 show that all PI films exhibit moderate thermal stability, with the 10% weight loss temperature (T_10_) of most films exceeding 410 °C, and most PIs showing a char yield of around 50% at 800 °C. The aromatic segments and rigid fused-ring scaffold of the diamine bearing the HEN skeleton endow the polymer backbone with good resistance to thermally triggered bond scission. Although the alicyclic HEN moiety inherently provides lower thermal stability than fully aromatic PIs, its rigid asymmetric fused-ring structure restricts high-temperature chain mobility and partially offsets the intrinsic weakness of alicyclic segments, ultimately governing the thermal degradation behavior of the present polyimides. DTG curves show weak weight-loss signals below approximately 350 °C, i.e., before the cumulative weight loss reaches 5%, attributed mainly to residual bound solvent and to the decomposition or volatilization of small amounts of low-molecular-weight oligomers and unreacted chain ends, rather than to incomplete imidization [28,29,30]. This assignment is consistent with the absence of amide and anhydride bands in the FT-IR spectra; because FT-IR is a qualitative technique, its detection limit does not allow residual amide/acid contents of a few weight percent to be excluded. The absence of these bands therefore confirms that imidization is complete in the bulk of the film, but it does not exclude trace uncyclized segments at chain ends. Although DMAc has a normal boiling point of approximately 166 °C, mass loss begins at around 250 °C because residual DMAc is hydrogen-bonded to amide/acid groups and trapped in the dense polymer matrix; below the glass transition (164–211 °C, Table 1) its diffusion is very slow, so measurable release sets in only above the glass transition, i.e., well above the boiling point of DMAc [31,32]. The maximum decomposition rate temperatures (T_max_) of the PI films range from 415 to 511 °C. These relatively high T_max_ values indicate that substantial main-chain scission only occurs at elevated temperatures, confirming the structural stability of the polymer backbones under high-temperature conditions. This primary DTG peak arises from preferential cleavage of the alicyclic HEN segments. PI 1–4 exhibit two well-resolved DTG peaks, whose secondary peaks, assigned to scission of the aromatic imide backbone, fall in the 547–619 °C range. For PI 5, the secondary degradation feature remains present yet becomes weak and broad, and is largely masked by the main decomposition peak. BPADA contains isopropylidene moieties that may be thermally labile. These groups may cleave within the temperature range of the primary decomposition peak and could promote early-onset scission of adjacent aromatic imide segments. This may shift aromatic imide backbone degradation toward lower temperatures and possibly produce considerable overlap between the two degradation temperature windows.

### 3.5. Hydrophobicity of PI Films

**Figure 5:**
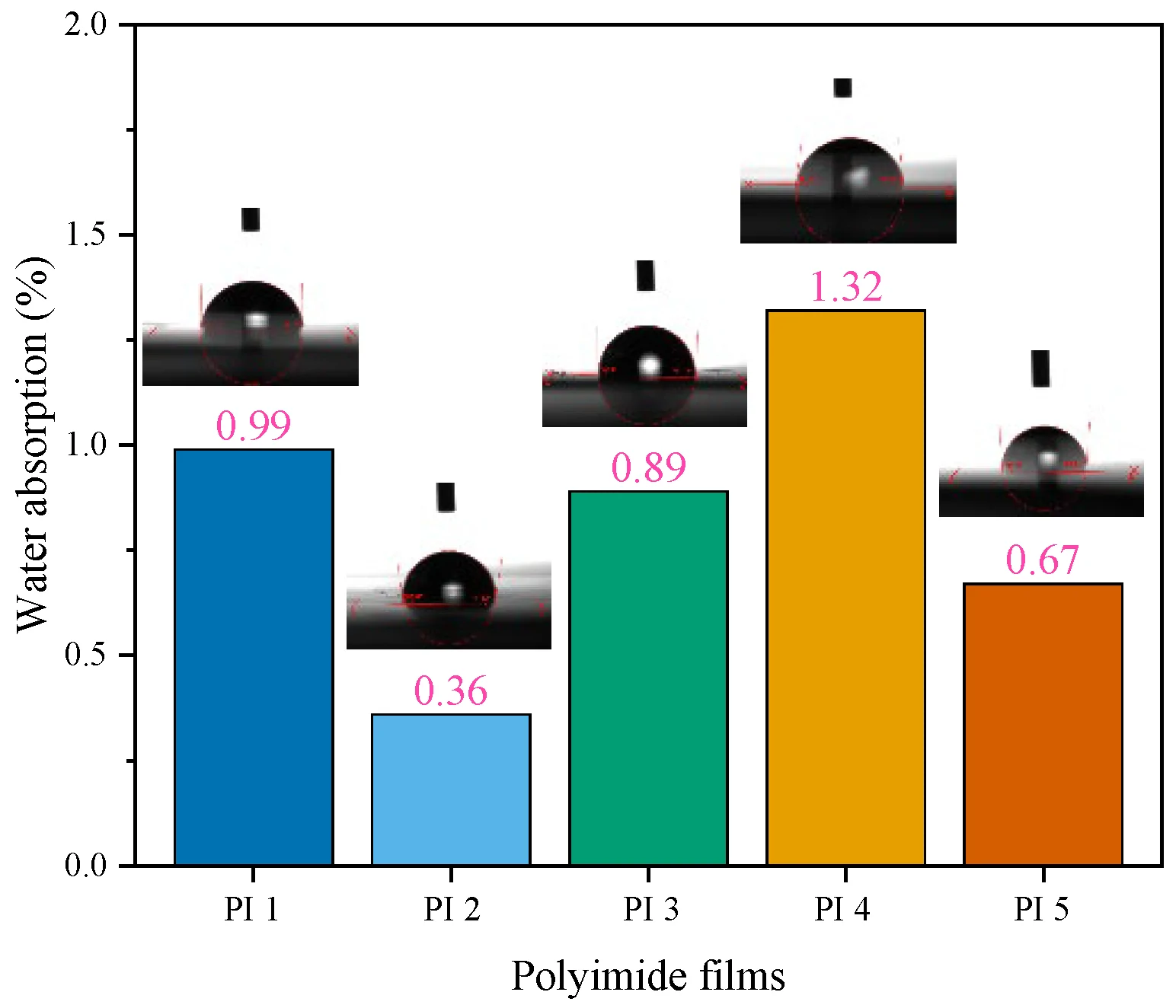
Water absorption and contact angle photographs of PI films.

Water absorption of PI 1–5 was determined via gravimetric measurement. Specimens of 3 × 3 cm were cut, weighed, and submerged in distilled water at 25 °C for 48 h. After immersion, the samples were removed, wiped dry, and reweighed. Water absorption values were calculated according to Equation (1). The experiment was repeated five times, and averaged results together with water contact angle photographs are presented in Figure 5, while the corresponding contact angles are summarized in Table 3.

(1) $$w=\frac{m_{2}-m_{1}}{m_{1}}$$

*w*: water absorption rate; *m*_1_: film mass before immersion; *m*_2_: film mass after immersion.

**Table 3:** Optical properties, thickness of PI films, calculated values for the frontier orbital energies and contact angle of PI films.

Codes	Optical Properties				Frontier Orbital Energies			CA/° ^d^
	Thickness/µm	T_450_/% ^a^	T_500_/% ^b^	λ_c_/nm ^c^	E_HOMO_/eV	E_LUMO_/eV	ΔE/eV	
PI 1	68	72.2	80.7	370	−5.42	−3.26	2.16	88.6
PI 2	77	65.5	78.7	378	−5.43	−3.31	2.12	98.6
PI 3	80	79.5	83.3	363	−5.38	−3.06	2.32	92.1
PI 4	82	32.6	59.9	388	−5.21	−3.63	1.58	83.3
PI 5	75	83.6	86.1	353	−5.34	−2.85	2.49	94.9

^a^ transmittance at 450 nm; ^b^ transmittance at 500 nm; ^c^ cutoff wavelength; ^d^ the contact angles of the PI films are reported as the average of five measurements.

Conventional aromatic polyimides readily absorb water because polar carbonyl and nitrogen sites on imide rings act as hydrogen-bond acceptors and interact with water molecules [33]. For example, Kapton and Upilex-S exhibit reported water absorption of approximately 2.7% and 2.5%, respectively [34]. The films in this work show lower water absorption (0.36–1.32%) than these conventional aromatic PIs, and their water contact angles (83.3–98.6°) are higher than those of literature-reported semi-alicyclic PIs from norbornene-containing dianhydrides (65.8–76.8°) [35]. The ENDAP-based polyimides are therefore more hydrophobic than both conventional aromatic and semi-alicyclic PIs, largely because the HEN skeleton and its pendant methyl groups lower molecular polarity and weaken water affinity [36]. Reduced molecular polarity and shielding of polar imide carbonyls weaken hydrogen bonding between water and imide groups and lower the thermodynamic driving force for water absorption [37].

PI 1 exhibits water absorption of 0.99 wt% and a contact angle of 88.6°. For comparison, the literature-reported BPDA/ODA polyimide, which contains the same BPDA dianhydride but the aromatic diamine ODA, shows a contact angle of only 79.6° [38]. This comparison suggests that the improved hydrophobicity mainly arises from reduced intrinsic water affinity originating from the ENDAP diamine moiety. PI 2 exhibits the best hydrophobic performance, with water absorption of 0.36% and a contact angle of 98.6°. This arises from trifluoromethyl groups in 6FDA, which possess low surface energy and suppress water adsorption at the polymer surface [39]. PI 4 absorbs the largest amount of water and shows the lowest contact angle, as conjugated carbonyl units from BTDA increase molecular polarity and strengthen interactions with water. PI 3 and PI 5 show water absorption of 0.89% and 0.67%, with contact angles of 92.1° and 94.9°, respectively. Both contain flexible ether linkages, and the ether groups are weak hydrogen-bond acceptors that may promote water sorption. In PI 5, however, the additional isopropylidene groups in BPADA lower molecular polarity and surface energy, and their steric hindrance reduces the exposure of polar imide carbonyls, further lowering water affinity. These effects outweigh those of the additional ether linkages, so PI 5 has more ether linkages than PI 3 but absorbs less water. Overall, the improved hydrophobicity relative to literature-reported PIs originates from the common ENDAP diamine, while the differences among PI 1–5 are governed mainly by the dianhydride structures.

### 3.6. Optical Performance of PI Films

**Figure 6:**
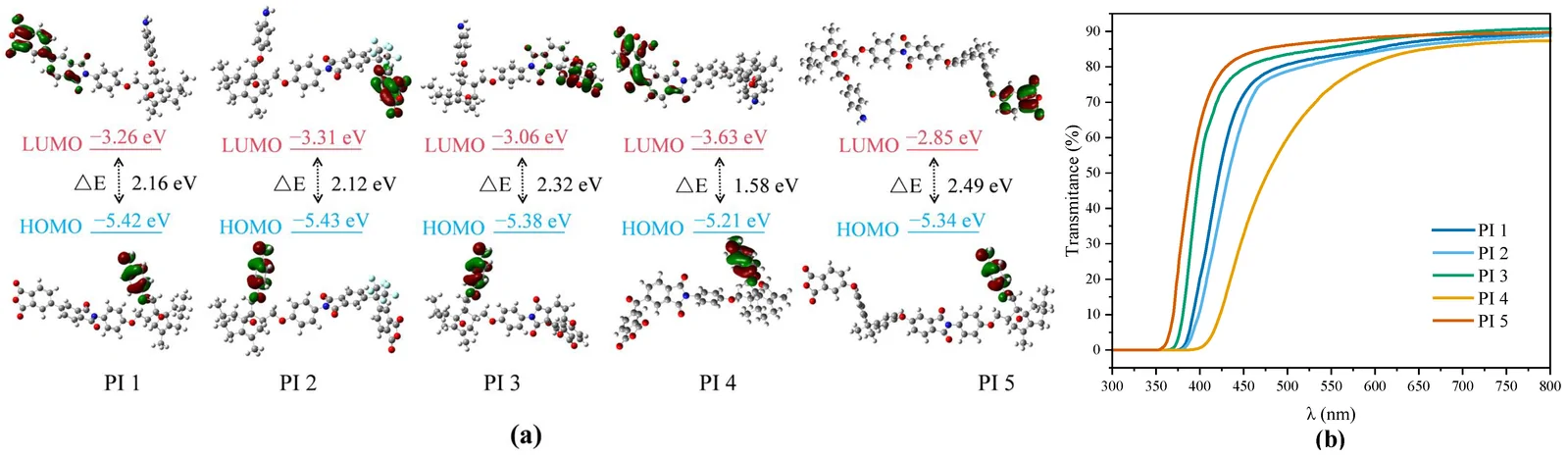
(**a**) DFT simulated molecular orbital energies and (**b**) UV–Vis spectra of PI films.

Conventional aromatic PIs tend to appear dark because of conjugated backbones and charge transfer (CT) between electron-donating diamine and electron-accepting dianhydride units. Disrupting conjugation and suppressing CT interactions improves optical transparency. Stronger backbone conjugation reduces the band gap (ΔE), induces red-shift of UV absorption and lowers visible transmittance, while a larger band gap corresponds to weaker CTC formation and higher optical transparency [40,41]. Frontier orbital energies and optical properties of this PI series were characterized to relate structure to transparency. The calculated orbital parameters are compiled in Table 3 and plotted in Figure 6a. A higher HOMO energy indicates stronger diamine nucleophilicity, whereas a lower LUMO energy indicates stronger dianhydride electrophilicity. UV–Vis spectra of the PI films with thicknesses reported in Section 2.5 were recorded. The transmittance at 450 nm (T_450_) and at 500 nm (T_500_), together with the ultraviolet cutoff wavelength (λ_c_), were obtained directly from the spectra. λ_c_ was defined as the wavelength at which the transmittance falls to the instrument baseline (near-zero transmittance). These parameters are summarized in Table 3, and the corresponding spectra are shown in Figure 6b.

Among the series, except PI 4, the films exhibit T_450_ of 65.5–83.6% and T_500_ of 78.7–86.1%. Across PI 1–5, λ_c_ ranges from 353 to 388 nm. Most of these PI films show good optical transparency, consistent with the HEN moiety in ENDAP. Its non-planar alicyclic structure, pendant methyl groups and asymmetric skeleton disrupt backbone planarity and regularity, restrict tight chain packing and π-π stacking, and reduce crystalline-domain light scattering [40,42]. These effects are expected to decrease the chain packing density and to enlarge the interchain d-spacing, thereby weakening intermolecular CTC interactions and providing a favorable optical baseline. The differences within the series, however, track the dianhydride structure. PI 4 contains the same ENDAP diamine but has the poorest optical performance, indicating that the dianhydride structure strongly modulates the final transparency and that the benefit of the HEN moiety can be counteracted by a dianhydride that promotes strong CTC. These structural effects are supported by previous WAXD tests, which show an enlarged interchain d-spacing for the present PIs compared with Kapton. DFT calculations reveal that ΔE follows the order PI 5 (2.49 eV) > PI 3 (2.32 eV) > PI 1 (2.16 eV) > PI 2 (2.12 eV) > PI 4 (1.58 eV). Consistent with the larger band gap–higher transparency relationship, this trend matches the optical performance obtained from UV–Vis tests.

PI 5 exhibits the best optical performance. It achieves the highest T_500_ of 86.1% and a T_450_ of 83.6%, with a λ_c_ of 353 nm. This optical behavior can be attributed to the ether linkage and the isopropylidene unit within BPADA. Both structural moieties disrupt backbone planarity, suppress backbone conjugation and reduce CTC formation, minimising visible light absorption. PI 1, PI 2 and PI 3 exhibit intermediate optical responses, with T_450_ of 72.2%, 65.5% and 79.5%, respectively. For PI 1, the rigid biphenyl structure delivers moderate backbone conjugation. For PI 2, bulky trifluoromethyl groups introduce steric repulsion to twist the polymer backbone and weaken conjugation. For PI 3, the flexible ether linkage interrupts the continuous conjugation pathway. These structural features result in moderate CTC interactions, and their band gap values accordingly fall between those of PI 5 and PI 4. In contrast, PI 4 exhibits the weakest optical performance. It has the lowest T_450_ and T_500_, reaching 32.6% and 59.9%, respectively, alongside the longest ultraviolet λ_c_ of 388 nm. Among the five dianhydrides used in this work, the benzophenone moiety in BTDA possesses the strongest electron-withdrawing capability and highest conjugation effect, which gives rise to the most pronounced CTC interactions, consistent with its smallest ΔE of 1.58 eV. This structural feature enhances polymer backbone conjugation and promotes CTC formation, shifting the UV absorption edge toward the short wavelength visible region and deteriorating optical transmittance. Compared with prior semi-alicyclic PIs constructed from norbornene-functionalized dianhydrides, which exhibited T_500_ of 19–53% and λ_c_ of 391–415 nm [35], the present films achieve higher T_500_ and shorter λ_c_, demonstrating the benefit of the HEN-based diamine for mitigating intermolecular CTC interactions. It can thus be seen that the HEN moiety originating from the diamine can substantially weaken intermolecular CTC interactions, yet cannot fully eliminate them. All these PIs also contain many aromatic units in the backbone, which absorb some short-wavelength visible light [3,4], leading to slight yellowing of most films. To limit further coloration, the final imidization temperature was kept at 230 °C, as higher temperatures caused noticeable film darkening. This may be attributed to more complete imidization and denser chain packing at higher temperatures, which enhance interchain CTC interactions, as well as possible thermal oxidation or crosslinking of the C=C double bond in the HEN unit, which could generate conjugated chromophores and red-shift the absorption edge [35].

## 4. Conclusions

In this work, a renewable asymmetric diamine ENDAP bearing a bulky, non-coplanar 3,4,5,6,7,8-hexahydro-2H-2,4a-epoxynaphthalene-3,4-diyl (HEN) alicyclic skeleton was synthesized from menthofuran and used to prepare a series of semi-alicyclic polyimide films. The HEN moiety is considered to contribute to reduced aromatic conjugation and perturbed chain packing regularity, thereby weakening intermolecular CTC interactions. Together with steric repulsion from its pendant methyl groups, these structural features are consistent with enlarged interchain d-spacing and fractional free volume, although the dianhydride structure also modulates these properties. The resulting PIs exhibit good solution processability in various organic solvents, with water absorption as low as 0.36%, glass transition temperatures ranging from 164 to 211 °C, and a maximum 10% weight-loss temperature of 451 °C under N_2_. The films exhibit λ_c_ of 353–388 nm and maximum T_450_ of 83.6% and T_500_ of 86.1%. The menthofuran-derived diamine is a useful renewable building block for semi-alicyclic polyimides. The resulting films combine good solubility, hydrophobicity, and optical transparency with acceptable thermal stability, making them interesting candidates for further study in microelectronic and optoelectronic applications.

## Figures and Tables

**Table 2:** Solubility of monomer and PI films.

Codes	CF	THF	EA	ET	DMF	TL	DMSO	DMAc	DCM	NMP
ENDAP	+	+	+	+h	+	+h	+	+	+	+
PI 1	+	±	–	–	+	–	+h	+	+	+
PI 2	+	±	±	–	+	–	+	+	+	+
PI 3	–	±	–	–	+	–	+	+	+	+
PI 4	±	±	–	–	±	–	±	±	±	+
PI 5	–	+h	–	–	+	–	+	+h	+	+

+: soluble at room temperature; –: insoluble; ±: slightly soluble at room temperature; +h: soluble after heating.

## Data Availability

The original contributions presented in this study are included in the article. Further inquiries can be directed to the corresponding authors.
